# Circulating microRNAs as Promising Biomarkers for Alzheimer's Disease: Insights from Human Plasma and Mouse Models

**DOI:** 10.1002/alz70856_103363

**Published:** 2025-12-25

**Authors:** Simran Yadav, Karolina Staniak, Aida Muntsant Soria, Lydia Giménez Llort, Urszula Wojda

**Affiliations:** ^1^ Laboratory of Preclinical Testing of Higher Standard, Nencki Institute of Experimental Biology of Polish Academy of Sciences, Warsaw, Poland; ^2^ Translational Behavioral Neuroscience Lab, Institut de Neurociències & Department of Psychiatry and Forensic Medicine, Universitat Autònoma de Barcelona, Barcelona, Spain

## Abstract

**Background:**

Identifying blood‐based biomarkers through cost‐effective, minimally invasive methods could revolutionize Alzheimer's disease (AD) diagnosis. While traditional markers such as Aβ and *p*‐tau remain well‐studied, circulating microRNAs (miRNAs) are emerging as promising alternative due to their stability in blood and abundance in the brain. Moreover, miRNAs can unveil complex AD molecular mechanisms. Our previous research identified several plasma miRNAs as potential AD biomarkers (Patent EP3449009, 2021; doi:10.1016/j.arr.2018.10.008; doi: 10.1016/j.arr.2018.10.008). However, systematic validation of these human biomarkers in AD animal models remains unexplored. This study aimed to analyze the levels of selected miRNA candidates associated with complex AD pathomechanisms: hsa‐miR‐29b‐3p, hsa‐miR‐146a‐5p, hsa‐miR‐200a‐3p, hsa‐miR‐483‐5p, and hsa‐miR‐486‐5p, in the blood plasma of the triple‐transgenic (3xTg‐AD) mouse model compared to control non‐transgenic counterparts (NTg).

**Method:**

Mice were housed in 4–5 per cage and maintained in standard laboratory animal conditions. Real‐time quantitative PCR (RT‐qPCR) was used to measure plasma miRNA levels in 16–20‐month‐old NTg and 3xTg‐AD mice, which exhibit well‐established AD‐like pathology in the brain. Each group consisted of 17 animals (8 females, 9 males). Spike‐in miR‐39‐3p served as an exogenous control to monitor RNA isolation and cDNA synthesis efficiency.

**Result:**

Endogenous miR‐192‐5p was identified as stable across all samples and used as a reliable normalizer for qPCR analysis. Significant differences in plasma miRNA levels of all investigated miRNAs were observed between NTg and 3xTg‐AD mice. In overall, alterations in miRNAs mirrored changes previously observed in human samples.

**Conclusion:**

The 3xTg‐AD mouse model exhibits plasma miRNA profile changes consistent with human miRNA molecular signature of AD, highlighting the potential of miRNAs as biomarkers for AD. This mice model also offers a valuable platform for studying the molecular mechanisms involving miRNAs in AD pathogenesis.

Supported by the Polish National Science Centre grant OPUS 2022/47/B/NZ7/03005 and the EU Horizon 2020 Research and Innovation grant no 737390 (ArrestAD).

